# Supplementary material for: Pregnancy reprograms the epigenome of mammary epithelial cells and blocks the development of premalignant lesions
Source: Nat Commun. 2020 May 27;11:2649. doi: 10.1038/s41467-020-16479-z (PMC7253414; doi:10.1038/s41467-020-16479-z)
Supplement: Supplementary file 1 — Supplementary Information [file 41467_2020_16479_MOESM1_ESM.pdf]

Supplementary Information

Title: Pregnancy reprograms the epigenome of mammary epithelial cells and blocks the development of premalignant lesions

Mary J. Feigman[1a], Matthew A. Moss[2a], Chen Chen[1a], Samantha L. Cyrill[1], Michael F. Ciccone[1], Marygrace C. Trousdell[1], Shih-Ting Yang[1], Wesley D. Frey[3], John E. Wilkinson[4], Camila O. dos Santos[1b]

Antibodies. All antibodies were purchased from companies as indicated below and used without further purification. Antibodies for lineage depletion: biotinylated anti-CD45 (eBioscience, #13-0451-85, 1:100 dilution), biotinylated anti-CD31 (eBioscience, #13-0311-85, 1:100 dilution), biotinylated anti-Ter119 (eBioscience, #13-5921-85, 1:100 dilution) and biotinylated anti-CD34 (eBioscience, #13-0341-82, 1:100 dilution). Antibodies for flow cytometry: eFluor 450 conjugated anti-CD24 (eBioscience, #48-0242-82, 1:100 dilution), PE-Cy7 conjugated anti-CD29 (eBioscience, #25-0291-82, 1:150 dilution), 7-AAD viability staining solution (BioLegend, #420404, 1:100 dilution), Alexa Fluor 405 conjugated anti-keratin 8/18 (Abcam, #ab210139, 1:300 dilution), Alexa Fluor 488 conjugated and β-casein (CSN2) (sc-166530, 1:300 dilution). Antibodies for negative controls: eFluor 450 conjugated mouse IgG (eBioscience, #48-4015-82, 1:100 dilution), FITC conjugated rat IgG (eBioscience, #11-4811-85, 1:300 dilution), and PE-Cy7 conjugated mouse IgG (BioLegend, #405315, 1:100 dilution). Antibody for MaSC enrichment: biotinylated anti-CD1d (BioLegend, #123505, 1:50 dilution). Antibodies for immunostaining (IF): Alexa Fluor 647 conjugated anti-keratin 5 (Abcam, #ab193895, 1:200 dilution), Alexa Fluor 405 conjugated anti-keratin 8/18 (Abcam, #ab210139, 1:200 dilution), Alexa Fluor 488 conjugated anti-cMYC (Abcam, #ab190026, 1:100 dilution), and Alexa Fluor 488 conjugated and β-casein (CSN2) (sc-166530, 1:300 dilution). Antibodies for Western Blot: anti-cMYC antibody (Y69, Abcam, #ab32072, 1:5000 dilution), anti-GAPDH (SCBT, #sc-365062, 1:5000 dilution), anti-p300 antibody (C-20, SCBT, #sc-585, 1:1000 dilution), anti-Acetyl-CBP (Lys1535/p300 Lys1499 Antibody CST, #4771, 1:1000 dilution), anti-STAT3 antibody (F-2, SCBT, #sc-8019, 1:500 dilution), anti-Vinculin antibody (EPR8185, Abcam, #ab129002, 1:5000 dilution), anti-p53 antibody (CM5, Leica Biosystems, #P53-CM5PL, 1:500 dilution), goat anti-rabbit IgG HRP (SCBT, #sc-2004, - 1:10.000 dilution), goat anti-mouse IgG HRP (Bio-Rad, #170-6516, - 1:20.000 dilution). Antibodies for ChIP-seq: anti-histone H3 (acetyl K27) antibody – ChIP grade (Abcam, #ab4729, 1:100 dilution). Antibodies for ChIP-qPCR and Cut&Run: anti-cMYC antibody (Abcam, #ab32072, 1:100 dilution).

Mammary gland isolation. Mammary glands were harvested and processed as previously described [1]. In short, mammary glands (four-five pairs per mouse) were harvested, minced and incubated for ~2 hours with 1x Collagenase/Hyaluronidase (10x solution, Stem Cell Technology) in RPMI 1640 GlutaMAX supplemented with 5% FBS. Digested mammary gland fragments were washed with cold HBSS supplemented with 5% FBS, followed by incubation with TrypLE Express (Thermo Fisher, #12604-013) and an additional HBSS wash. Cells were incubated with 2 mL of Dispase (Stem Cell Technology) supplemented with 40 μL DNAse I (Sigma, #D4263) for 2 minutes and then filtered through a 100μm Cell Strainer (BD Falcon, #352360). The single cell

suspension was incubated with lineage depletion antibodies and loaded onto MACS magnetic column (Miltenyi Biotech). Flow-through cells (epithelial cells) were utilized for protein isolation, flow cytometry, transcriptomic and epigenomic analysis. For experiments described on Fig.1 mammary glands harvested from 5 pre- or post-pregnancy female mice, or 3 1st or 2nd EPH, were pulled together as one biological replicate per molecular profile experimental task (RNA-seq, H3K27ac ChIP-se), and from 2 mice as one biological replicate per mammary fatpad transplantation and mammary organoid cultures. For experiments described on Fig.2 mammary glands harvested from 1 mouse were pulled together as one biological replicate per experimental task (RNA-seq, H3K27ac ChIP-seq, cMYC Cut&Run). For experiments described on Fig.3 mammary glands harvested from 2 mice were pulled together as one biological replicate per experimental task (mammary fatpad transplantation and mammary organoid cultures). For experiments described on Fig.4, mammary glands harvested from 1 mouse were pulled together as one biological replicate per experimental task (RNA-seq, H3K27ac ChIP-seq, ATAC-seq, cMYC Cut&Run, and mammary organoid cultures).

Mammary Organoid Culture. Mammary glands were dissected from nulliparous and parous Balb/C female mice (Fig.1) or CAGMYC female mice (Fig.3,4), minced and digested for ~40 minutes in Collagenase A, type IV solution, following a series of centrifugations to enrich for mammary organoids [2]. For experiments presented on Fig.1, freshly isolated mammary organoids were cultured for 6 days with Essential media (Advanced DMEM/F12, supplemented with ITS (Insulin/Transferrin/Sodium selenite, Gibco, #41400-045, and FGF-2 (PeproTech, #450-33)) prior to analysis. Medium was changed every day. Culture mammary organoids were then grown with FGF-2 depleted or Complete media (Advanced DMEM/F12, supplemented with ITS (Insulin/Transferrin/Sodium selenite, Gibco, #41400-045, Prolactin (Sigma, #L4021), 17βEstradiol (Sigma #E2758) and Progesterone (Sigma, #P8783)). For experiments described on Figure 3, pre- and post-pregnancy freshly isolated CAGMYC organoids were cultured with Essential media for 2 days, and treated with doxycycline (DOX, 0.5mg/mL) for 1 (DD1) and 2 days (DD2). For experiments described on Figure 4, pre- and post-pregnancy CAGMYC organoids were cultured with Essential media for 2 days, then cultured with Essential media and doxycycline (DOX, 0.5mg/mL) for 2 (DD2) or 5 days (DD5), and treated with either cMYC inhibitor (cMYCi = 10058-F4, 5μM, Calbiochem, #475956) or Histone Acetyltransferase inhibitors (HATi II, 10μM, AOBIOUS, #AOB4032; PU139 20μM, AOBIOUS, #AOB0257). Cell visualization and image collection was performed on a Nikon Eclipse TI microscope utilizing NIS-Elements BR software (Nikon). At least 10 fields of view from at least 2 independently conducted experiments were analyzed for organoid size and branching quantification. The area (size) of focused mammary

organoids in each image was measured by ImageJ, and the organoid with three or more elongated buds was scored as "branching". Statistically significant differences for organoid branching were considered with Student t-test *p*-value lower than 0.05 ($p<0.05$).

Flow cytometry. Lineage depleted (Lin-), Luminal ($CD24^{high}CD29^{low}$) and Myoepithelial MECs ($CD24^{+}CD29^{high}$) harvested from female mice were sorted using a FACS ARIAII SORP (BD Bioscience). Organoids cultures were isolated from Matrigel (Corning 354230) using 0.5ml Cell Recovery solution (Corning 354253) and digested using 0.5ml Gibco TrypLE (12604-013) for 10 minutes at 37°C. Tripsin digestion was quenched with 1ml of Essential media, following pipetting until single cell suspension was achieved (~20 times). Single cells are then fixed in 1% PFA for 20 minutes at room temperature and permeabilized using Invitrogen eBioscience™ Intracellular Fixation & Permeabilization (88-8824-00). Cells were stained for 30 minutes with indicated antibodies (1:300 dilution) in Invitrogen 1x Perm/wash buffer. For cell analysis, Dual Fortessa II cell analyzer (BD Bioscience) was used. Data analysis was performed using Diva 8 Software (BD) or FlowJo (Tree Star). Statistically significant differences for ductal quantification were considered with Student t-test *p*-value lower than 0.05 ($p<0.05$).

Pregnancy hormone exposure analysis. Female mice were implanted with 21 days-slow-release estrogen and progesterone pellets (17β-Estradiol (0.5 mg/pellet) + Progesterone (10 mg/pellet) – Innovative Research of America), or placebo control pellets as previously described [1]. Nulliparous Balb/C female mice (never pregnant) and parous Balb/C female mice (1 natural pregnancy cycle) were implanted with slow-release pellets and mammary glands were examined at days 6 and day 12 post pellet implantation.

Mammary fat pad transplantation. CD1d+MaSCs from pre- and post-pregnancy Balb/C female mice (Fig.1) or pre- and post-pregnancy CAGMYC female mice (Fig.3) were isolated as previously described [1, 3]. In short, lineage depleted MECs were incubated with biotinylated anti-CD1d antibody, followed by MACS magnetic column enrichment. CD1d-enriched MaSCs were ressuspended with 50% growth factor reduced matrigel solution (BD Biosciences) and injected into the cleared fat-pad of the inguinal mammary gland (anterior part of the gland). Transplanted mammary glands were harvested ductal quantification. Error bars indicate standard error of mean across samples of same experimental group. Statistically significant differences were considered with Student t-test *p*-value lower than 0.05 ($p<0.05$). For experiments presented on Fig.1 and Fig.3d-f, Balb/C CD1d-enriched MaSCs (~1K) were injected into the mammary fatpad of 15-days old Balb/C female mice, and allowed for a period of 8-weeks for tissue engraftment prior to exposure to pregnancy hormones (6 days, Fig.1) or DOX-treatment for 5 days (Fig.3d-f). For

experiments presented on Fig.3g, pre- or post-pregnancy CAGMYC CD1d-enriched MaSCs (~100K) were injected into the mammary fatpad of 8-10 weeks old CAG-only female mice, and allowed 3-days of tissue engraftment prior to DOX-treatment for 30 days. We used transgenic CAG-only female mice as hosts for the transplantation assays to avoid immune rejection due to transgene expression [4].

Histological analysis: For histological analysis, the left inguinal mammary gland was harvested and fixed in 4% PFA overnight prior to paraffin embedding. For conventional histological analysis, mammary gland tissue slides were stained with Hematoxylin and Eosin (H&E). Images were acquired using Aperio ePathology (Leica Biosystems) slide scanner in 40X lenses. For Immunofluorescence staining (IF), paraffin-embedded mammary gland sections were deparaffinized in Xylene (Sigma, #534056) and rehydrated, followed by antigen retrieval in Trilogy (Cell Marque, 920P-10). Tissue was washed in 1x PBS (phosphate-buffered saline) for 1 min then blocked with blocking solution (10mM Tris-HCl pH 7.4, 100mM $MgCl_2$, 0.5% Tween 20, 10% FBS, 5% goat serum) for 4 hours in a humidified chamber. Sections were stained with the appropriate conjugated primary antibodies in blocking solution for 16 hours at 4°C. After subsequent washings with 1x PBS and blocking solution, slides were mounted in ProLong with DAPI (Invitrogen). Cell visualization and image collection was performed on a Zeiss LSM780 confocal laser-scanning microscope utilizing Zen lite software, Blue edition, version 2.0.0.0 (Zeiss). For ductal quantification, mammary gland H&E histological images were uploaded into Image J, and only ducts present in the posterior part of the gland (half) were manually counted. Statistically significant differences for ductal quantification were considered with Student t-test *p*-value lower than 0.05 ($p<0.05$).

Doxycycline (DOX) treatment. Doxycycline was purchased from Takara Bio USA, Inc. (#631311) and sucrose was purchased from Sigma (S7903). DOX drinking solution (1 mg/mL) was prepared using sterile 1% sucrose water. CAGMYC female mice and all control mice were given DOX water for 5 days (DD5) or otherwise specified.

RT-PCR and qPCR analysis. For RT-qPCR analysis, mammary organoids were homogenized in TRIzol (Thermo Fisher Scientific, #15596018) for RNA extraction. Double stranded cDNA was synthesized from purified RNA using SuperScript III Reverse Transcriptase (Thermo Scientific). qPCR primers (Supplementary Table 1) were utilized for quantitative PCR reactions on QuantStudio 6 real time PCR system, Software v1.3 (Thermo Fisher) and quantification results were analyzed using the delta delta CT method. Statistically significant differences were considered with Student t-test *p*-value lower than 0.05 ($p<0.05$). Error bars indicate standard error

of mean across samples of same experimental group. n=3 biological replicates and 6 technical replicates. For ChIP-qPCR, pre- and post-pregnancy Lineage negative (Lin^-) MECs were used for chromatin pulldowns using antibodies specific for cMYC (Abcam, #ab32072) and rabbit IgG (Millipore, #12-371). ChIP samples were analyzed by quantitative PCR reactions on a Quantstudio 6 real time PCR system (Thermo Fisher), utilizing primers designed across the transcription start site of the genes Epha2 and Tbx3 (Supplementary Table 2). Results are displayed as fraction of Input (non-immunoprecipitated DNA). n=2. Error bars indicate standard error of mean across samples of same experimental group. Statistically significant differences were considered with Student t-test *p*-value lower than 0.05 ($p<0.05$). Error bars indicate standard error of mean across samples of same experimental group. n=2 biological replicates and 6 technical replicates for Epha2 measurements and n=3 technical replicates for Tbx3 measurements.

Western blot. MECs from untreated or DOX-treated (DD5) pre- or post-pregnancy CAGMYC transgenic mice were isolated and homogenized in 1x Laemmli sample buffer (Bio-Rad). Samples were loaded into homemade 10% SDS-Page gel and transferred overnight to PVDF membrane using wet-transfer apparatus (Bio-Rad). Membranes were blocked with 1% BSA solution and incubated overnight with a diluted solution of primary antibody, followed by incubation with HRP-conjugated antibody for 40 minutes. HRP signal was developed with Luminata Crescendo Western HRP substrate (Millipore) in autoradiography film (Lab Scientific, #XARALF2025). Developed films were scanned on Epson Perfection 2450 photo scanner.

RNA-seq library preparation and analysis. MECs were collected and homogenized in TRIzol LS (Thermo Fisher Scientific, #10296010) for RNA extraction. Double stranded cDNA synthesis and Illumina libraries were prepared utilizing the Ovation RNA-seq system (V2) (Nugen Technologies, #7102-32). RNA-seq libraries were prepared utilizing the Ovation ultralow DR multiplex system (Nugen Technologies, #0331-32). Each library (n=2 per experimental condition) was barcoded with Illumina True-seq adaptors to allow sample multiplexing, followed by sequencing on an Illumina NextSeq500, 76bp single-end run. We used STAR [5] for mapping reads. We used DESeq [6] to assess changes in expression levels simultaneously across multiple conditions and in multi-factor experimental designs, incorporating information from multiple replicates (2 independent experiments per cell type). Gene Set Enrichment Analysis (GSEA) was used for global analysis of differentially expressed genes [7, 8]. Further downstream analyses were performed using various R packages, including gplots for heatmaps [9], base R [10], and ggplots2 for other visualizations [11]. For experiments presented on Fig.1, FACS-sorted, lineage

depleted, luminal MECs were isolated from: Balb/C female mice pre-pregnancy (5 mice), post-pregnancy (5 mice), first pregnancy cycle at day 6 (D6) and day 12 (D12) (3 mice per time point), second pregnancy cycle at day 6 (D6) and day 12 (D12) (3 mice per time point). For experiments presented on Fig.2, lineage depleted total MECs were isolated from DOX–treated, pre-pregnancy, CAGMYC female mice at day 2 (DD2) and at day 5 (DD5) (2 mice per treatment). For experiments presented on Fig.4, FACS-sorted, lineage depleted luminal and myoepithelial MECs were isolated from DOX–treated CAGMYC pre-pregnancy at day 5 and DOX–treated CAGMYC post-pregnancy at day 5 (DD5) (2 females per experimental group).

Mammary tumor tissue gene expression analysis. For the analysis presented on Fig.2d and Supplementary Fig.3b, a total of 157 mammary tumor tissue samples were retrieved from publicly available datasets (GEO numbers GSE13221, GSE15904,GSE30805), and preprocessed using the 'affy' R package [12], in order to obtain gene expression levels. Batch effect normalization of CAGMYC RNA-seq and publicly available microarray data was performed using the ComBat function from the 'sva' R package [13]. Principal component analysis was done using the base R principle component function in order to project all gene expression data into a low dimensional space.

ChIP-seq library preparation and analysis. Formaldehyde-fixed lineage depleted MECs OT FACS-isolated MECs were used for chromatin pulldowns using antibody specific for H3K27ac histone marks (Abcam, #ab4729). Each library (n=2 per experimental condition, with the exception for pre-pregnancy CAGMYC DD2, with has 1 replicate) was amplified and barcoded using Clontech DNA Smart ChIP-Seq kit (Clontech, #634866) in accordance with the manufacturer's instructions, then pooled for sequencing on an Illumina NextSeq500, 76bp single-end run. Reads were mapped to the indexed mm9 genome using bowtie2 short-read aligner tool [14], using default settings. MACS2 peak-calling program [15] was used to identify enriched genomic regions in this data by comparing the pulldown ChIP data to the control (Input) data using a tag size of 25bp and a q-value cutoff of 1.00^{-3} (Fig.1), or a q-value cutoff of 1.00^{-2} (Figs. 2 and 4). The ROSE (Rank Order of Super-Enhancers) algorithm [16] was used to identify enhancers and super-enhancers throughout the genome using ChIP-seq BAM files and GFF files. GFF files were made from MACS-called BED files. ROSE analysis was performed using default conditions, which included a 2.5 Kb exclusion zone surrounding the TSS and a 12.5 Kb stitching distance. The UCSC genome browser was used to analyze genomic regions for overlap, using the Bedtools intersect function. Any overlap between regions (enhancers) was considered "shared", whereas and no overlap between regions (enhancers) defined the regions as ONLY being in one sample.

The comparison was made into a venn diagram using tool available at https://www.meta-chart.com/venn). Identification of genes closest to these differentially called enhancer regions was preformed using Genomic Regions Enrichment of Annotations Tool (GREAT) [17]. Homer's annotatePeaks.pl script was run with default settings in order to define genomic distributions. The heatmap of the peak intensities was produced using the plotHeatmap tool in deepTools2 [18]. Peak visualizations were generated using the UCSC Genome Browser [19]. ebox DNA motifs were defined using the fimo command from the Meme Software Suite. For experiments presented on Fig.1, FACS-sorted luminal MECs were isolated from: Balb/C female mice pre-pregnancy (5 mice), post-pregnancy (5 mice), first pregnancy cycle at day 6 (D6) and day 12 (D12) (3 mice per time point), second pregnancy cycle at day 6 (D6) and day 12 (D12) (3 mice per time point). For experiments presented on Fig.2, lineage depleted total MECs were isolated from DOX–treated, pre-pregnancy, CAGMYC female mice at day 2 (DD2) and at day 5 (DD5) (2 mice per treatment). For experiments presented on Fig.4, FACS-sorted, luminal and myoepithelial MECs and lineage depleted total MECs were isolated from DOX–treated CAGMYC pre-pregnancy at day 5 and DOX–treated CAGMYC post-pregnancy at day 5 (DD5) (4 mammary glands from 2 females were pulled together in each experimental group).

ATAC-seq library preparation and analysis. Nuclei of lineage depleted total MECs from nulliparous and parous CAGMYC females treated with DOX (DD5) were isolated utilizing hypotonic lysis buffer and incubated with Tn5 enzyme from Nextera DNA sample Preparation kit (Illumina, #FC-121-1031) for the preparation of ATAC libraries. Each library (n=2 per experimental condition) were amplified and barcoded as previously described [20], then pooled for sequencing on an Illumina Nextseq500, 76bp single-end run. ATACseq library reads (n=2 per cell condition) were mapped to the indexed mm9 genome using Bowtie short read-aligner [14]. MACS2 [15] was used was used to identify enriched genomic regions in this data using a tag size of 25bp and a q-value cutoff of 1.00^{-2}. Peaks were annotated using Homer with standard mm9 genome references [21]. Location of peaks was then grouped into intergenic, promoter and genic (containing 5'UTR, Exons, Introns, Transcription Termination Sites, 3'UTR, ncRNA, miRNA, snoRNA, and rRNA) regions. The UCSC genome browser was used to analyze genomic regions for overlap, using the Bedtools intersect function. Any overlap was enough to consider two regions "shared" and no overlap, between the genomic regions defined the regions as exclusively being in one sample. The comparison was made into a venn-diagram using tool available at https://www.meta-chart.com/venn).

Cut & Run library preparation and analysis. Lineage depleted total MECs from nulliparous and parous CAGMYC females treated with DOX (DD5) were isolated, and Cut&Run chromatin

profiling was performed as previously described [22]. In short, MECs were permeabilized with digitonin, following incubation with cMYC antibody for 12 hours. Antibody-chromatin complexes were fragmented with pA-MNAse and purified utilizing Phenol-Chloroform. Cut & Run libraries (n=2 for pre-pregnancy CAGMYC MECs and n=3 for post-pregnancy CAGMYC MECs) were amplified and barcoded using Clontech DNA Smart ChIP-Seq kit (Clontech, #634866) in accordance with the manufacturer's instructions, then pooled for sequencing on an Illumina NextSeq500, 76bp paired-end run. Reads were mapped to the indexed mm9 genome using bowtie2 short-read aligner tool [14], using default settings. Sparse Enrichment Analysis for CUT&RUN (SEACR) peak-calling program [23] was used to identify enriched genomic regions, with an empirical threshold of n=0.01, returning the top n fraction of peaks based on total signal within peaks. The stringent argument was implemented which used the summit of each curve. To compute differentially bound sites, DESeq2 [24] was utilized to generate genomic regions that are statistically significantly, differentially bound by cMYC between sample groups (FDR<0.05).

cMYC Cut&Run DNA motif analysis. Pre- and post-pregnancy CAGMYC cMYC Cut&Run peaks were utilized as input for DNA motif-based sequence analysis. In short, peak sequences were uploaded into Find Individual Motif Occurrences (FIMO) [25], to identify sequences of known motifs, with a statistical threshold of 0.0001.

Supplementary Figure 1

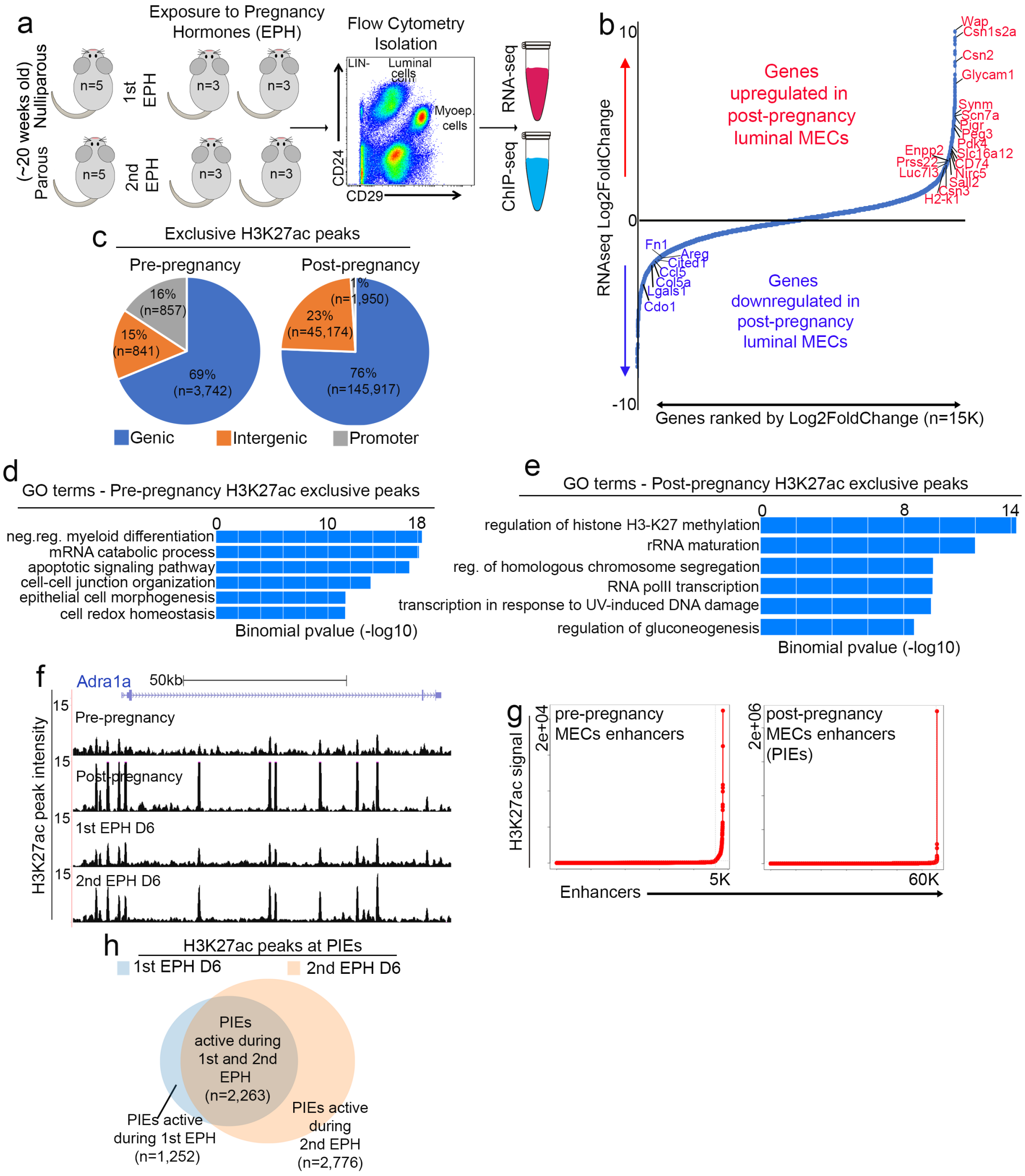

Characterization of the molecular state of pre- and post-pregnancy MECs. **(a)** Experimental strategy for epigenetic and transcription analysis of luminal MECs harvested from female mice spanning diverse development stages. (**b**) Differential expression of previously defined parity-induced signature (Log2FoldChange) in luminal MECs harvested from nulliparous and parous female mice. Red arrow = Log2FoldChange greater than 2; Blue arrow = Log2FoldChange lower than 2. Red font = genes upregulated in post-pregnancy MECs; Blue font= genes downregulated in post-pregnancy MECs. (**c**) Classification of exclusive H3K27ac peaks of pre- and post-pregnancy luminal MECs according to their genomic location (genic, intergenic or promoter region). **(d)** Gene Ontology (GO) term analysis of H3K27ac peaks exclusive to pre-pregnancy MECs. (**e**) Gene Ontology (GO) term analysis of H3K27ac peaks exclusive to post-pregnancy MECs. (**f**) Genome browser tracks showing distribution of H3K27ac peaks at distinct pregnancy cycles for Adra1a locus. (**g**) ROSE classification of H3K27ac peaks from pre- and post-pregnancy MECs into enhancer regions. (**h**) Venn-diagram comparing H3K27ac peaks at PIEs in luminal MECs harvested during first and second EPH (exposure to pregnancy hormone).

Supplementary Figure 2

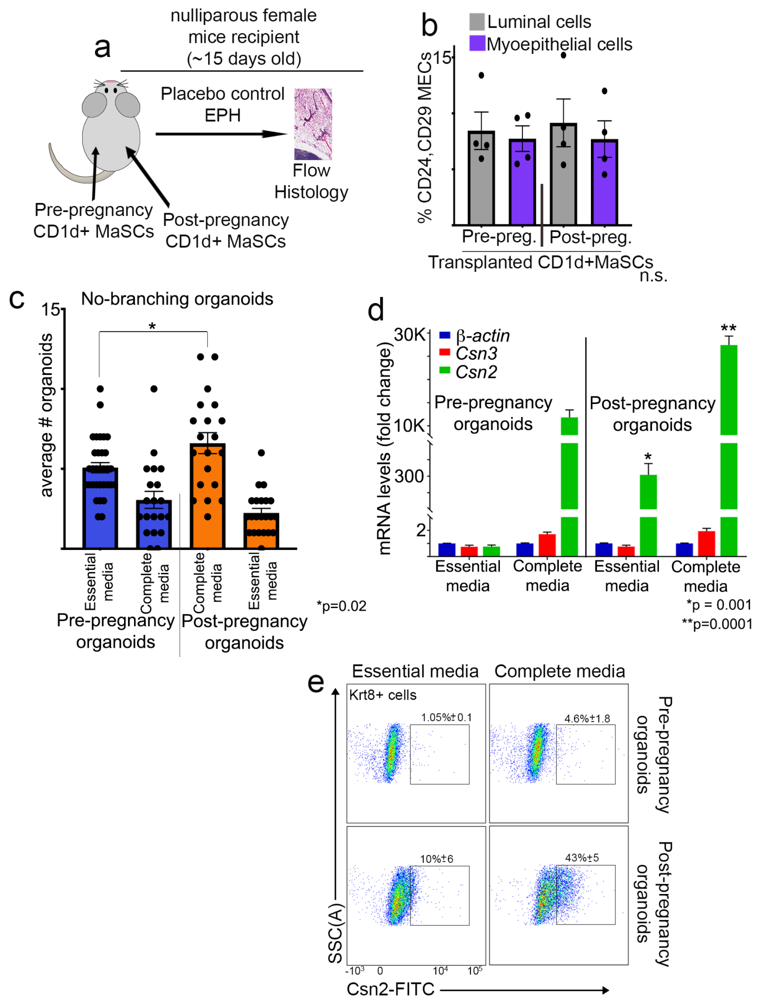

Exposure to Pregnancy Hormone (EPH) on MEC development. (**a**) Scheme of experimental design utilized for MaSCs transplantation, and tissue analysis. n=3 mammary glands injected with pre-pregnancy CD1d+ MaSCs, and n=3 mammary glands injected with post-pregnancy CD1d+ MaSCs. (**b**) FACS quantification of MECs from pre- or post-pregnancy CD1d+ MaSCs transplants (8 weeks post-transplantation). n=4 mammary glands injected with pre-pregnancy CD1d+ MaSCs, and n=4 mammary glands injected with post-pregnancy CD1d+ MaSCs. n.s.= not statistically significant. (**c**) Representative images and branching quantification of mammary organoid culture derived from pre- and post-pregnancy MECs, grown with either Essential media or Complete media (containing Estrogen (E2), Progesterone (P4) and prolactin (Prol)). n=3 independent biological replicates, *p=0.02 and **p=0.003. Scale: 200 µm. (**c**) Quantification of non-branched mammary organoid culture derived from pre- and post-pregnancy MECs, grown with either Essential or Complete media. n=3 independent biological replicates, *p=0.02. Scale: 200 µm. (**d**) qPCR quantification of casein genes (Csn2 and Csn3) in pre- and post-pregnancy mammary organoid cultures, grown with either Essential or Complete media. n=2 independent biological experiments with 3 technical replicates each. *p=0.001 = differences between pre- and post-pregnancy mammary organoids grown with either Essential or Complete media; **p=0.0001 = differences between pre- and post-pregnancy mammary organoids grown with pregnancy hormones. (**e**) Intracellular flow cytometry analysis of CSN2 protein levels in pre- and post-pregnancy mammary organoid cultures, grown with either Essential or Complete media. CSN2 intracellular protein levels were quantified in pre-gated KRT8+ cells. n=2 biological replicates, with 2 technical replicates each. For all analysis, error bars indicate standard error of mean across samples of same experimental group. p-values were defined using Student t-test.

Supplementary Figure 3

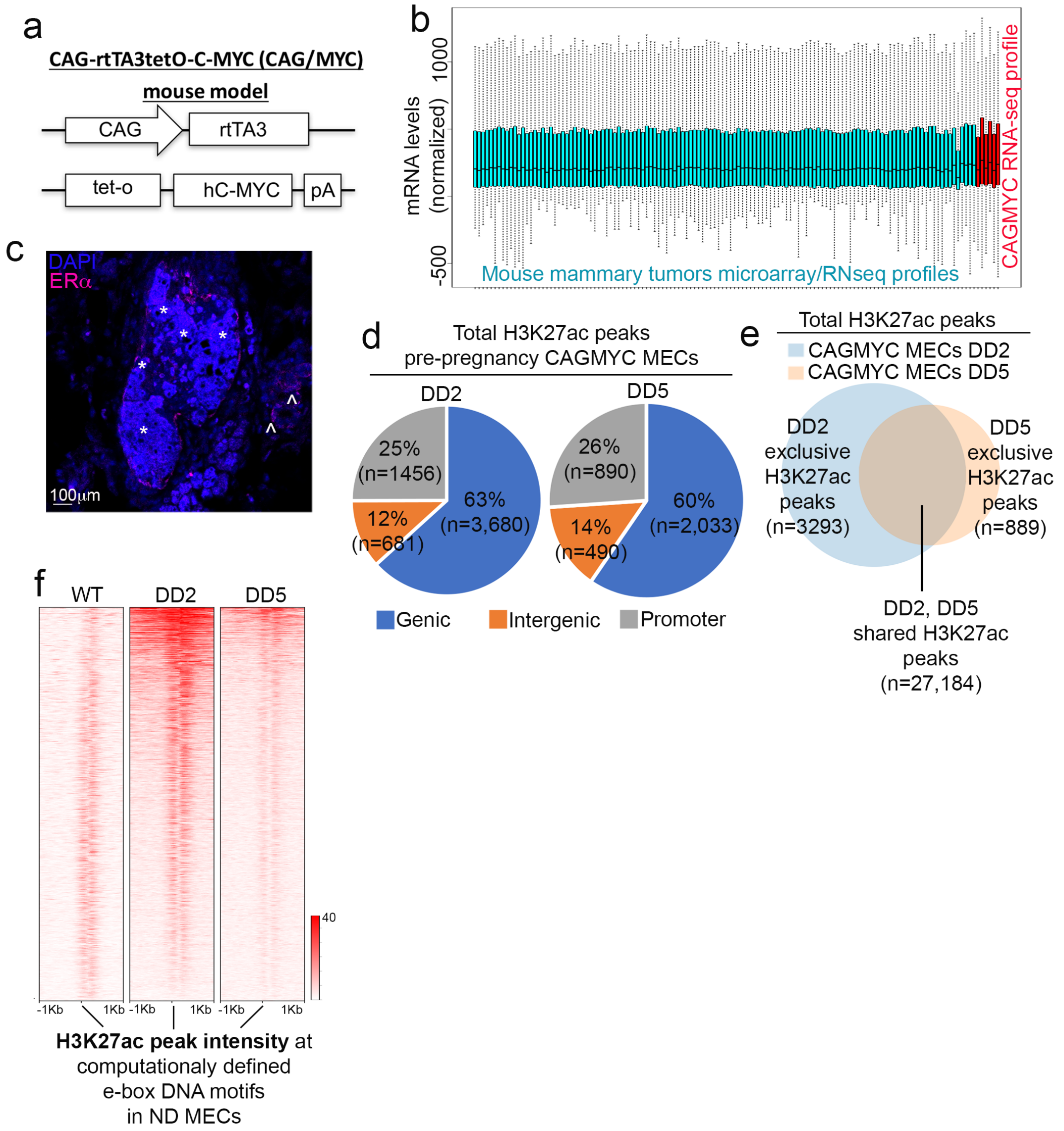

***cMYC* overexpression drives mammary premalignant lesions.** (**a**) Diagrammatic representation of the transgenic mouse strain CAGMYC showing alleles for overexpression of human *cMYC*, driven by the CAG promoter, under the inducible control of the Tet operator-repressor system. (**b**) Boxplots from Combat batch effect normalization analysis on gene expression profiles from expression microarrays (publicly available data) and RNA-seq (CAGMYC), demonstrating similar dynamic range of expression across datasets after normalization. (**c**) Immunofluorescence image of mammary gland from CAG-only transplanted with pre-pregnancy CAGMYC CD1d+ MaSCs, demonstrating areas of ERα/DAPI co-staining (*) as indication for ERα nuclear staining, and areas where ERα does not overlap with DAPI signal (^), indicating areas of ERα cytoplasmic staining. Scale: 100 μm. (**d**) Classification of total H3K27ac peaks from pre-pregnancy CAGMYC MECs treated with DOX for 2 days (DD2, left) and 5 days (DD5, right), according to their genomic distribution. (**e**) Venn-diagram comparing H3K27ac peaks between pre-pregnancy CAGMYC MECs treated with DOX for 2 days (DD2) and 5 days (DD5). (**f**) Density plot showing computationally defined e-box DNA binding motifs with high H3K27ac peak intensity in MECs harvested from WT nulliparous female, compared to H3K27ac peak intensity at same e-box DNA binding sites from MECs from CAGMYC female mice treated with DOX for 2 days (DD2) and 5 days (DD5).

Supplementary Figure 4

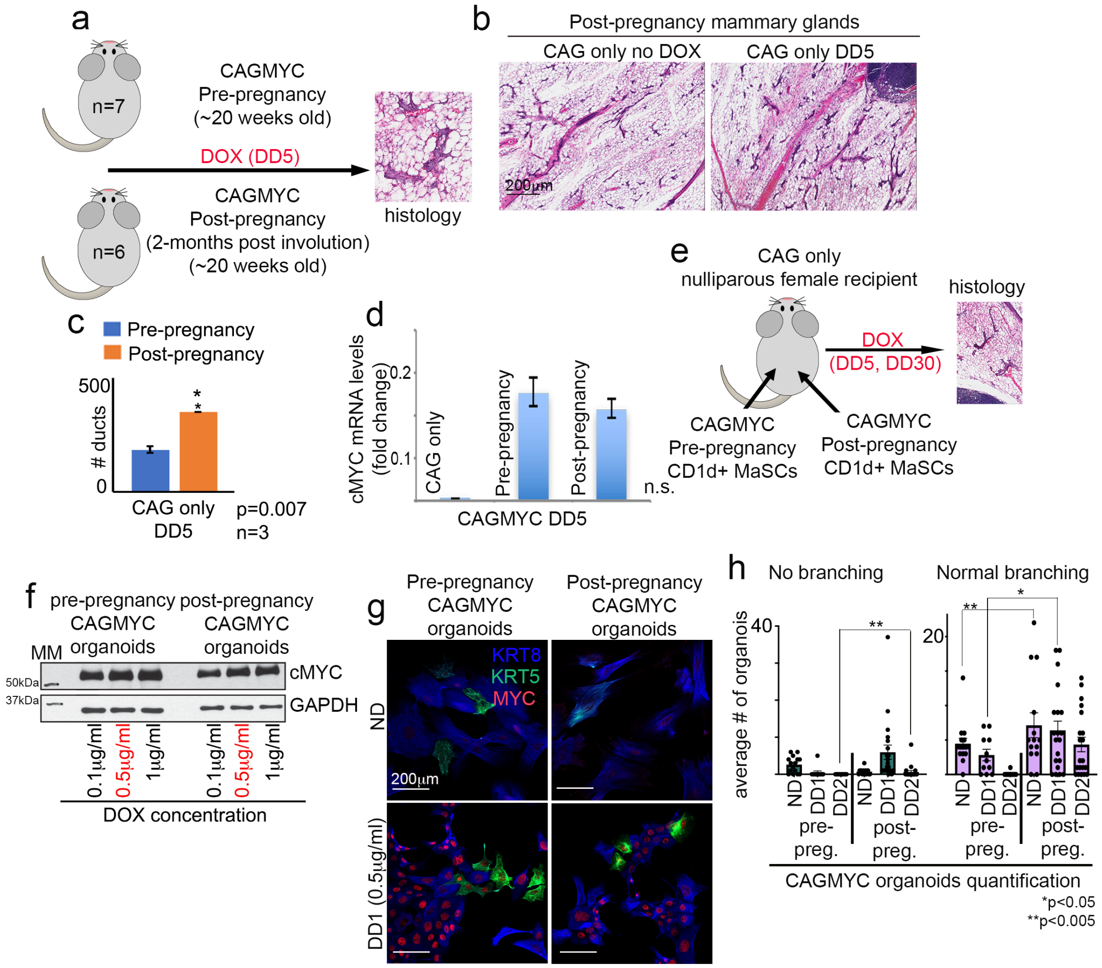

The effects of *cMYC* overexpression on post-pregnancy mammary glands. (a) Experimental strategy for inducing *cMYC* overexpression in nulliparous (n=7 animals) and parous (n=6 animals) CAGMYC female mice by DOX treatment (DD5). (**b**) H&E stained mammary gland images from DOX-treated, parous female mice lacking *cMYC* transgenic allele (CAG-only). (**c**) Quantification of ductal structures in from nulliparous and parous CAG only transgenic female mice after DOX treatment (DD5). Bars indicate mean number of ducts. n=3 DOX-treated, pre-pregnancy CAG-only mammary glands, and n=3 DOX-treated, post-pregnancy CAG-only mammary glands were analyzed. *pvalue= 0.007. (**d**) Quantification of human *cMYC* mRNA levels by qPCR in control (CAG only) and in pre- and post-pregnancy CAGMYC MECs treated with DOX for 5 days. Bars indicate mean log2 fold change. n= 2 biological replicates, with a total of 6 technical replicates. n.s. = not statistically significant. (**e**) Experimental strategy for analysis of nulliparous CAG only female mice transplanted with CAGMYC CD1d+ MaSCs, followed by DOX treatment for 5 days (DD5) and 30 days (DD30). (**f**) Western blot analysis of *cMYC* and GAPDH levels in pre- and post-pregnancy CAGMYC organoid cultures treated with several concentrations of DOX. Red-highlighted concentration (0.5μg/ml) indicates DOX concentration utilized in all experiments presented in the manuscript. MM = Molecular Marker. (**g**) Immunofluorescence image of pre-pregnancy and post-pregnancy CAGMYC MECs harvested from organoid cultures and treated with DOX for 1 day (DD1), visualizing DAPI (blue), KRT5 (green), cMYC (yellow). Scale: 200 μm. (**h**) Average of total pre- and post-pregnancy CAGMYC organoid cultures, classified into No branching (**p<0.005) and Branching (*p<0.05 and **P<0.005), before and after induction of *cMYC* overexpression (DOX treatment). For all analysis, error bars indicate standard error of mean across samples of same experimental group. p-values were defined using Student t-test.

Supplementary Figure 5

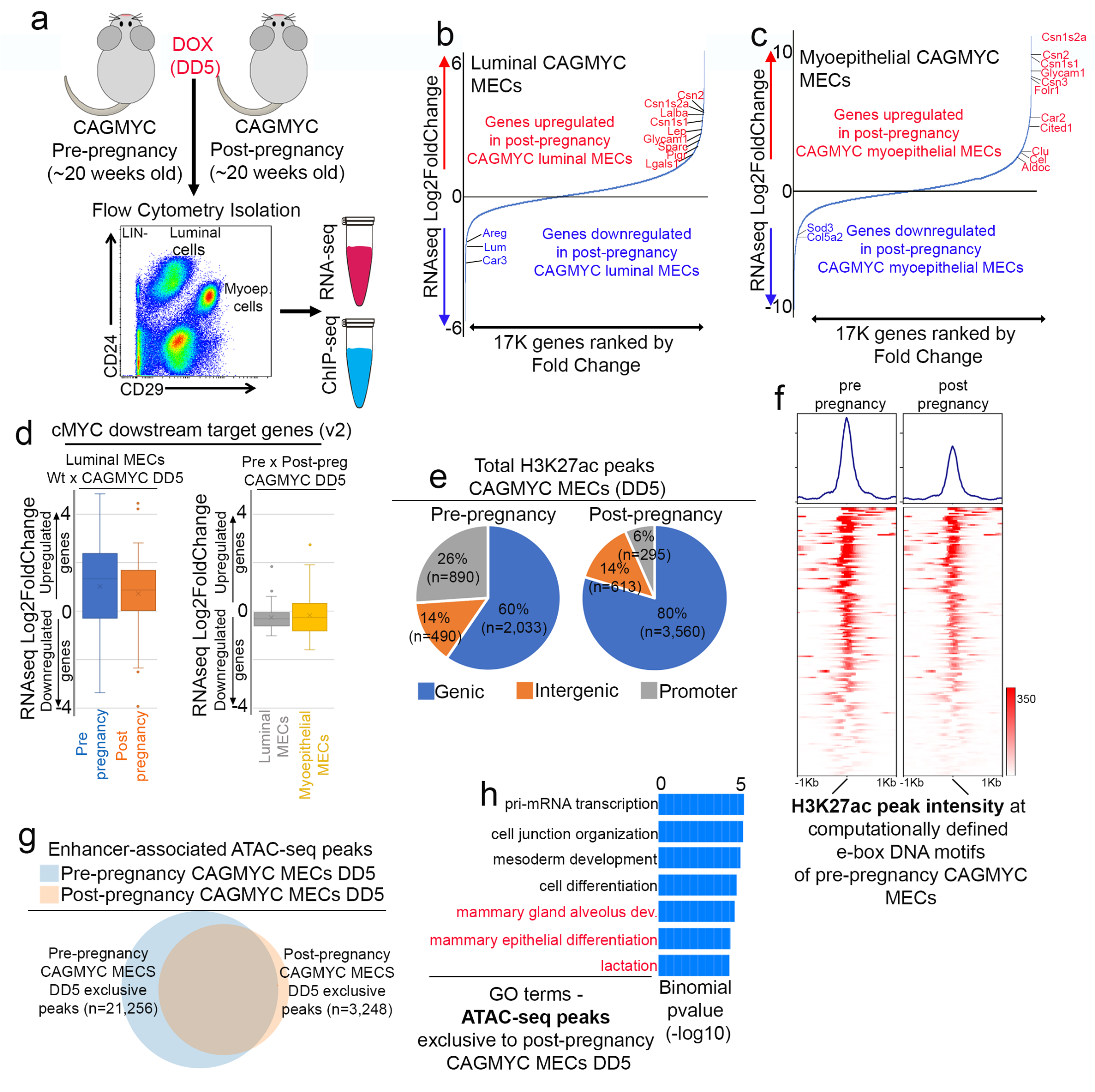

Characterization of cMYC-induced molecular changes in post-pregnancy MECs. (a) Scheme of experimental strategy for epigenomic and transcriptomic analysis of isolated MECs from CAGMYC female mice after DOX treatment (DD5). n=2 mice per library preparation. n=2 libraries per analysis. (**b-c**) Differential expression of previously defined parity-induced signature (Log2FoldChange) in luminal (b) and myoepithelial (c) MECs harvested from nulliparous and parous CAGMYC female mice (DD5). Red arrow = Log2FoldChange greater than 2; Blue arrow = Log2FoldChange lower than 2. Red font= genes upregulated in DD5 post-pregnancy CAGMYC MECs; Blue font= genes downregulated in DD5 post-pregnancy CAGMYC MECs. (**d**) Differential RNA expression (Log2Foldchange) of MYC downstream targets gene expression analysis, comparing the effects of cMYC overexpression (CAGMYC DD5) over non-transgenic MECS (WT) in pre- and post-pregnancy luminal MECs (left panel), and the effects of cMYC overexpression in pre- and post-pregnancy CAGMYC MECs (luminal and myoepithelial). Center line represents the median of the dataset. (**e**) Classification of total H3K27ac peaks from pre- and post-pregnancy CAGMYC MECs treated with DOX for 5 days (DD5), according to their genomic distribution. (**f**) Density plot showing computationally defined e-box DNA binding motifs with high H3K27ac peak intensity in MECs harvested from CAGMYC nulliparous female (DD5), compared to H3K27ac peak intensity at same e-box DNA binding site in MECs from CAGMYC nulliparous female (DD5). (**g**) Venn diagram showing unique and shared ATAC-seq peaks in pre- and post-pregnancy CAGMYC MECs treated with DOX for 5 days (DD5). (**h**) Gene Ontology (GO) term analysis of ATAC-seq peaks exclusive of DD5 post-pregnancy CAGMYC MECs.

Supplementary Figure 6

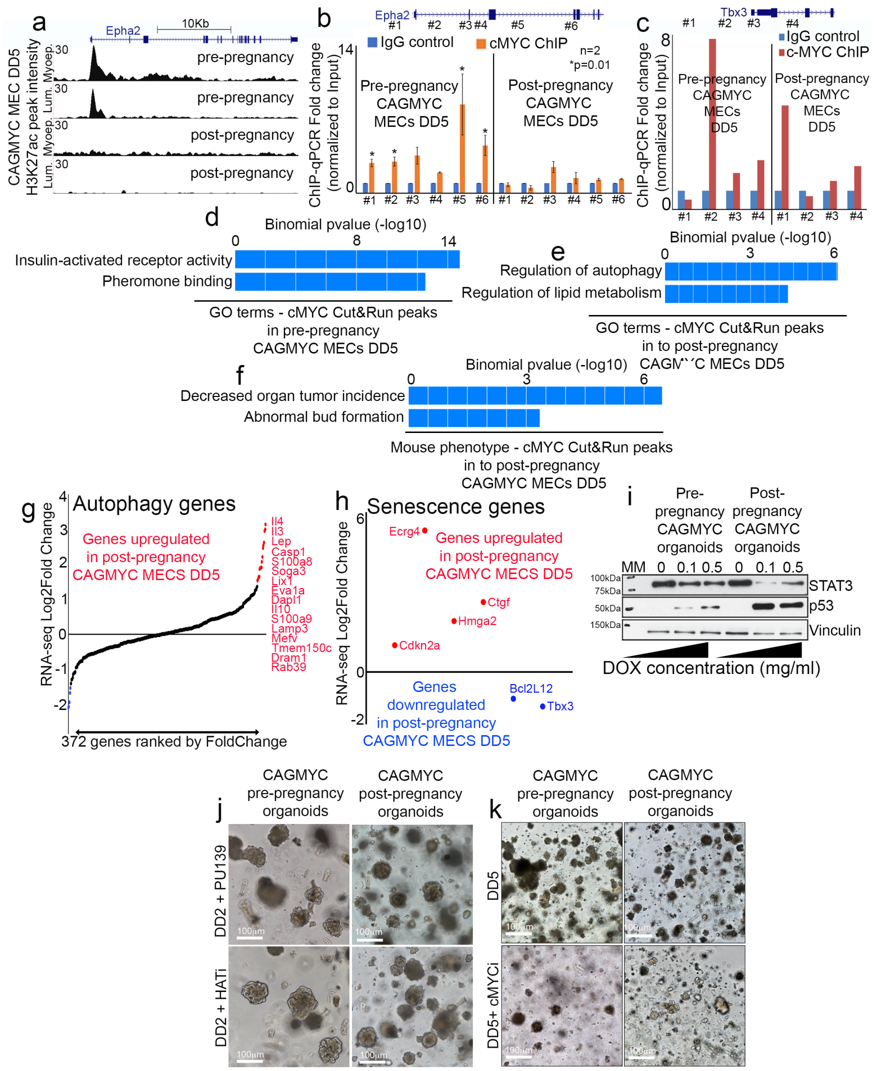

cMYC occupancy is reduced in post-pregnancy CAGMYC MECs. (a) Genome Browser tracks for EphA2 gene in DOX-treated, FACS-isolated, pre- and post-pregnancy CAGMYC MECs. (**b-c**) ChIP-qPCR quantification of cMYC occupancy at the EphA2 gene locus (**b**) and Tbx3 gene locus (**c**) in DOX-treated, pre- and post-pregnancy CAGMYC MECs. Bars indicate fold enrichment in relation to Input. (**b**) n=2 biological replicates and 6 technical replicates. *p=0.01. (**c**) n=3 technical replicates. (**d**) Gene Ontology (GO) term analysis enriched in cMYC peaks exclusive of DD5 pre-pregnancy CAGMYC MECs. (**e**) Gene Ontology (GO) term analysis enriched in cMYC peaks exclusive of DD5 post-pregnancy CAGMYC MECs. (**f**) Molecular Phenotype term analysis enriched in cMYC peaks of post-pregnancy CAGMYC MECs. (**g-h**) Differential expression of (g) autophagy associated genes and (h) senescence associated genes (Log2FoldChange) in luminal MECs harvested from nulliparous and parous CAGMYC female mice, DD5. Red = genes upregulated in post-pregnancy MECs; Blue = genes downregulated in post-pregnancy MECs. (**i**) Western blot of showing STAT3 and p53 protein levels in organoid cultures derived from pre-pregnancy and post-pregnancy CAGMYC MECs, with and without DOX treatment (2 days). Vinculin protein levels was used as endogenous control. MM = Molecular Marker. (**j-k**) Representative images of mammary organoid culture of pre- and post-pregnancy CAGMYC MECs, grown with Essential media and DOX (2 days, 0.5μg/mL), with and without Histone Acetyltransferase inhibitors (HATi II, 10μM, and PU139, 20μM) (j) or with and without cMYC inhibitor (cMYCi, 10058-F4, 10μM). Error bars indicate standard error of mean across samples of same experimental group. p-values were defined using Student t-test.

Supplementary Table 1. Primer list for genes utilized in RT-qPCR analysis.

Gene	Forward	Reverse
Csn2	CTGTGCTCCGCCTCATAAA	GAGAAGAAACCAGGTGAGTCTG
Csn3	TCAGGATAACACCGCCATTC	TCAGGATTGGCCACAGTATTT
Gaphd	CCCATCACCATCTTCCAGGAG	CTTCTCCATGGTGGTGAAGACG
βactin	GGCTGTATTCCCCTCCATCG	CCAGTTGGTAACAATGCCATGT

Supplementary Table 2. Primer list for genes utilized in ChIP-qPCR analysis.

Gene	Forward	Reverse
mEpha2_#1	GCT ACG GAA GCT TCT ACT TCT C	CAC CAG GGT TAG TGT CCA AAT A
mEpha2_#2	GCA CCT TCC ACT TCC TAA CTA C	TCC TGA CCT CTG TCA CAT CTA T
mEpha2_#3	AGG CCA ACA ACC CAT TCT TAT	CAT AGA ACA CCC TGA CCC AAT C
mEpha2_#4	GCA CAA TGC ACC CAT GAT AAG	AGG CCA TGA CTC AGG ATA GA
mEpha2_#5	TGT ACC AAG TCA GGG TCA TTT C	GTT TCC TGA CCA GCC TTC TT
mEpha2_#6	CAG GAC GGT GTA GAG ACT ATG A	AAG GAA ATG GGC GAA ACT AGA A
mTbx3_#1	CTG CCT CTC TCT GTC TCT CTT T	CCC ACA TCC CTC TTT CTT CAA TAC
mTbx3_#2	CAG GGA AAG AGC GTA GGT TTC	GGA CAG GGA AAC GGG TAA TAA G
mTbx3_#3	CTT AAT CTC TAG GCC CGG AAA C	GGA GGG CAA CTT AGC AGA AT
mTbx3_#4	CCC TTG CCT GAC CGA AAT AA	CAA TGC TAC CTA CCT CTG GTT T

Supplementary Table 3. cMYC Cut&Run Transcription Factor DNA motif analysis

Total number of cMYC Cut&Run peaks	Number of peaks with motif occurrence (p-value=0.0001)	% of Input peaks	Transcription Factor (TF)	p-value	q-value	Motif sequence
Virgin: 28,089	7,741	27.56 %	cMYC	9.5e-07	0.177	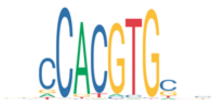
Parous: 35,452	6,634	18.71 %	cMYC	8.67e-07	0.263	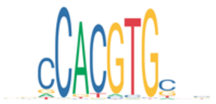

Supplementary References

1. dos Santos, C.O., et al., *Molecular hierarchy of mammary differentiation yields refined markers of mammary stem cells.* Proc Natl Acad Sci U S A, 2013. **110**(18): p. 7123-30.
2. Nguyen-Ngoc, K.V., et al., *3D culture assays of murine mammary branching morphogenesis and epithelial invasion.* Methods Mol Biol, 2015. **1189**: p. 135-62.
3. Frey, W.D., et al., *BPTF Maintains Chromatin Accessibility and the Self-Renewal Capacity of Mammary Gland Stem Cells.* Stem Cell Reports, 2017. **9**(1): p. 23-31.
4. Hanahan, D., *Transgenic mouse models of self-tolerance and autoreactivity by the immune system.* Annu Rev Cell Biol, 1990. **6**: p. 493-537.
5. Dobin, A., et al., *STAR: ultrafast universal RNA-seq aligner.* Bioinformatics, 2013. **29**(1): p. 15-21.
6. Anders, S. and W. Huber, *Differential expression analysis for sequence count data.* Genome Biol, 2010. **11**(10): p. R106.
7. Mootha, V.K., et al., *PGC-1alpha-responsive genes involved in oxidative phosphorylation are coordinately downregulated in human diabetes.* Nat Genet, 2003. **34**(3): p. 267-73.
8. Subramanian, A., et al., *Gene set enrichment analysis: a knowledge-based approach for interpreting genome-wide expression profiles.* Proc Natl Acad Sci U S A, 2005. **102**(43): p. 15545-50.
9. Warnes, G., Bolker, B., Bonebakkler, L., Gentleman, R., Huber, W., Liaw, A., Lumley, T., Maechler, M., Magnusson, A., Moeller, S., Scwartz, M., Venebles, B. *gplots: Various R Programming Tools for Plotting Data. R package version 3.0.1.* 2016; Available from: https://CRAN.R-project.org/package=gplots.
10. Team, R.C. *A language and environment for statistical computing.* 2018; Available from: https://http://www.R-project.org/.
11. Maag, J.L.V., *gganatogram: An R package for modular visualisation of anatograms and tissues based on ggplot2.* F1000Res, 2018. **7**: p. 1576.
12. Gautier, L., et al., *affy--analysis of Affymetrix GeneChip data at the probe level.* Bioinformatics, 2004. **20**(3): p. 307-15.
13. Leek, J.T., et al., *The sva package for removing batch effects and other unwanted variation in high-throughput experiments.* Bioinformatics, 2012. **28**(6): p. 882-3.
14. Langmead, B. and S.L. Salzberg, *Fast gapped-read alignment with Bowtie 2.* Nat Methods, 2012. **9**(4): p. 357-9.
15. Zhang, Y., et al., *Model-based analysis of ChIP-Seq (MACS).* Genome Biol, 2008. **9**(9): p. R137.
16. Loven, J., et al., *Selective inhibition of tumor oncogenes by disruption of super-enhancers.* Cell, 2013. **153**(2): p. 320-34.
17. McLean, C.Y., et al., *GREAT improves functional interpretation of cis-regulatory regions.* Nat Biotechnol, 2010. **28**(5): p. 495-501.
18. Ramirez, F., et al., *deepTools2: a next generation web server for deep-sequencing data analysis.* Nucleic Acids Res, 2016. **44**(W1): p. W160-5.
19. Dreszer, T.R., et al., *The UCSC Genome Browser database: extensions and updates 2011.* Nucleic Acids Res. **40**(Database issue): p. D918-23.
20. Buenrostro, J.D., et al., *Transposition of native chromatin for fast and sensitive epigenomic profiling of open chromatin, DNA-binding proteins and nucleosome position.* Nat Methods, 2013. **10**(12): p. 1213-8.
21. Heinz, S., et al., *Simple combinations of lineage-determining transcription factors prime cis-regulatory elements required for macrophage and B cell identities.* Mol Cell, 2010. **38**(4): p. 576-89.
22. Skene, P.J. and S. Henikoff, *An efficient targeted nuclease strategy for high-resolution mapping of DNA binding sites.* Elife, 2017. **6**.

23. Meers, M.P., D. Tenenbaum, and S. Henikoff, *Peak calling by Sparse Enrichment Analysis for CUT&RUN chromatin profiling.* Epigenetics Chromatin, 2019. **12**(1): p. 42.
24. Love, M.I., W. Huber, and S. Anders, *Moderated estimation of fold change and dispersion for RNA-seq data with DESeq2.* Genome Biol, 2014. **15**(12): p. 550.
25. Cuellar-Partida, G., et al., *Epigenetic priors for identifying active transcription factor binding sites.* Bioinformatics, 2012. **28**(1): p. 56-62.
