# Supplementary material for: Pregnancy reprograms the epigenome of mammary epithelial cells and blocks the development of premalignant lesions
Source: Nat Commun. 2020 May 27;11:2649. doi: 10.1038/s41467-020-16479-z (PMC7253414; doi:10.1038/s41467-020-16479-z)
Supplement: Supplementary file 4 — Source Data [file 41467_2020_16479_MOESM4_ESM.zip › Feigman&Moss&Chen_DataSource/Feigman&Moss&Chen_DataSource_Westernblots.pdf]

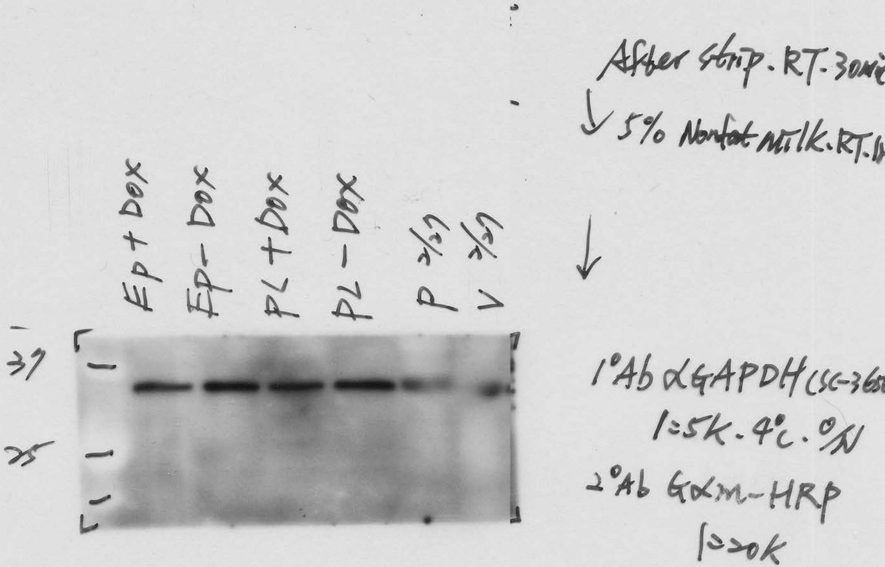

Feigman & Moss & Chen - GAPDH western blot - Fig3

15 2018/3/25

Feigman & Moss & Chen - cMYC western blot - Fig.3

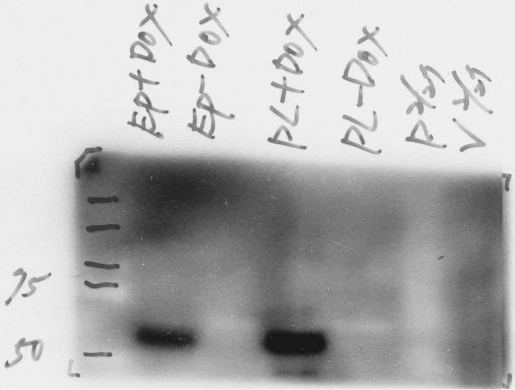

10% SDS-PAGE
P3 20V 7 mins transfer
PVDF

56kDa 49kDa
1°Ab α cMyc (Y69) ab32072
1:10k · 4c O/N

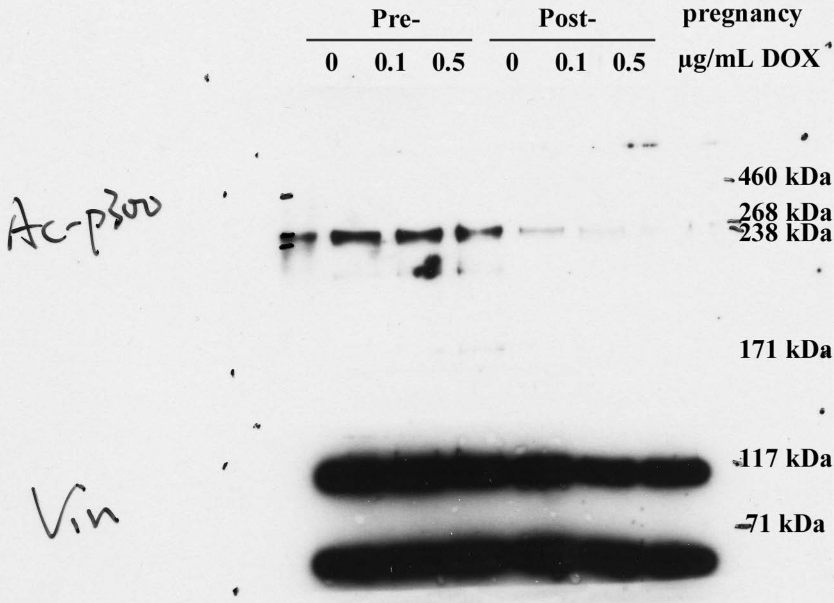

Feigman & Moss & Chen - acp300 western blot - Fig.4h

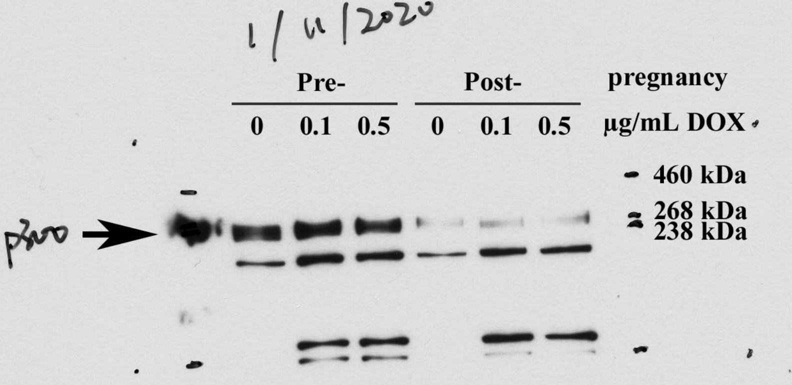

Feigman & Moss & Chen - p300 western blot - Fig.4h

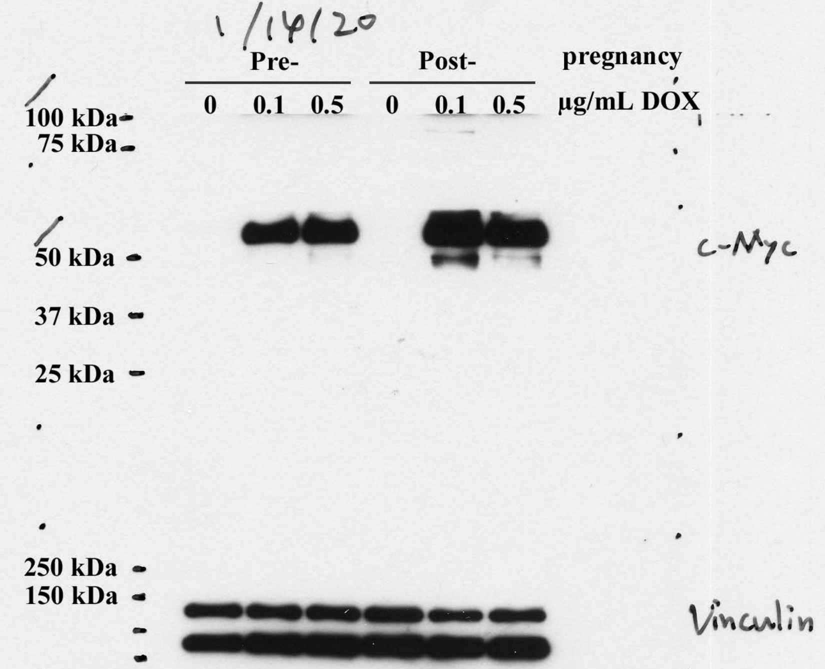

Feigman & Moss & Chen - vinculin western blot - Fig.4h

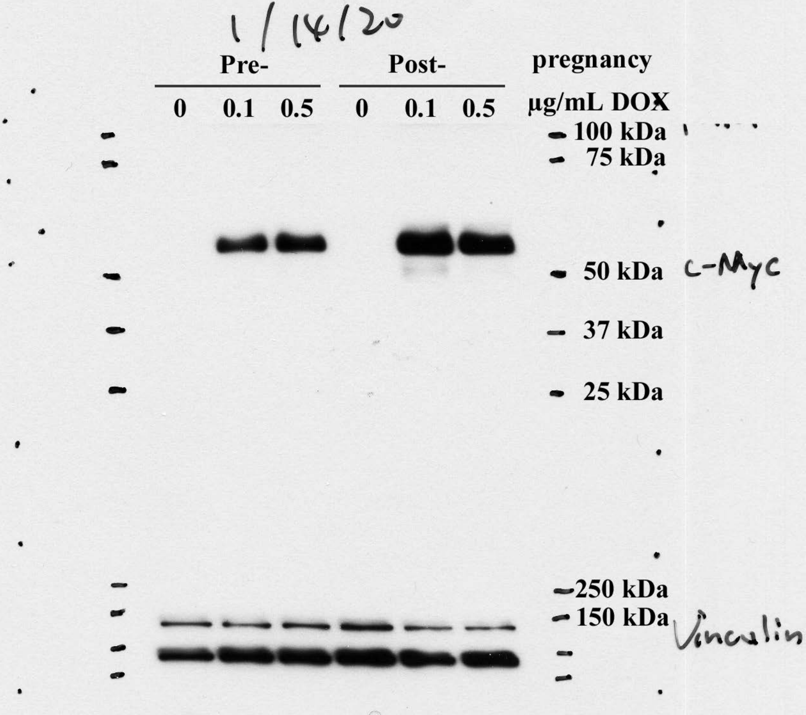

Feigman & Moss & Chen - cMYC western blot - Fig.4h

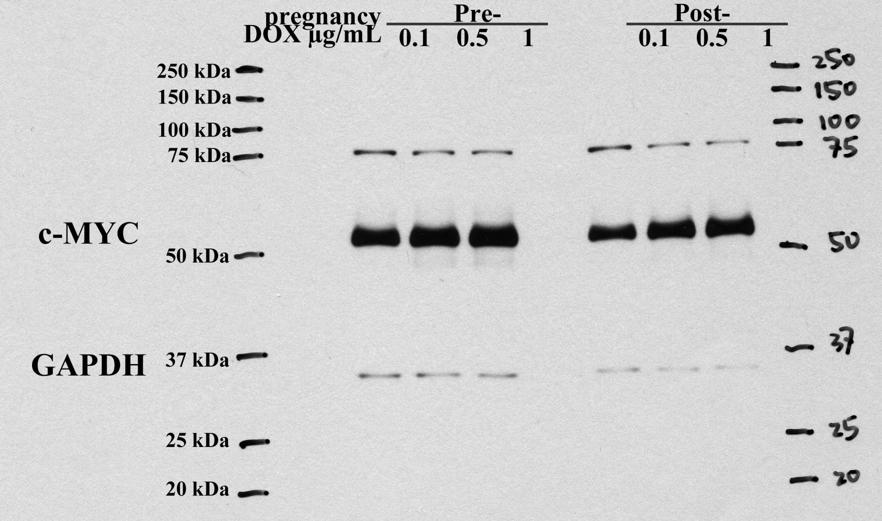

Feigman & Moss & Chen - cMYC western blot - Sup.Fig.4f

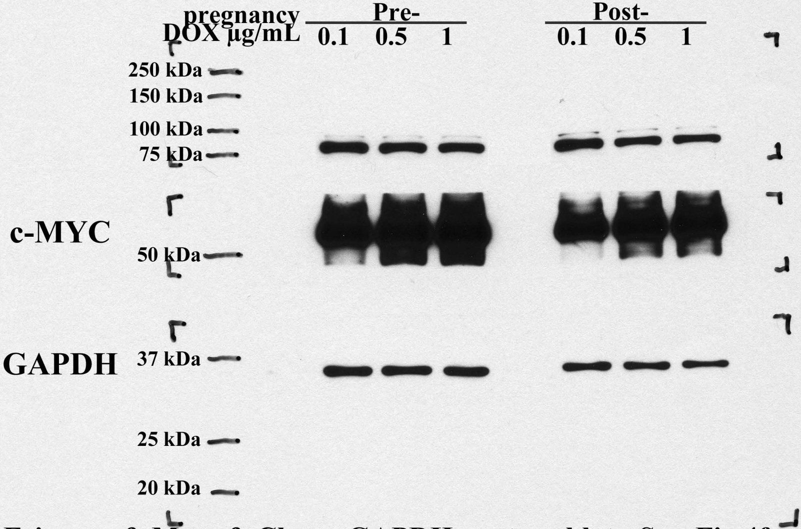

Feigman & Moss & Chen - GAPDH western blot - Sup.Fig.4f

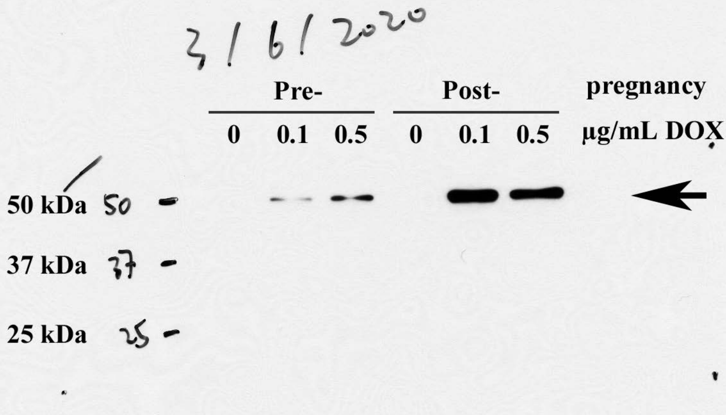

Feigman & Moss & Chen p53 western blot - Sup.Fig.6i

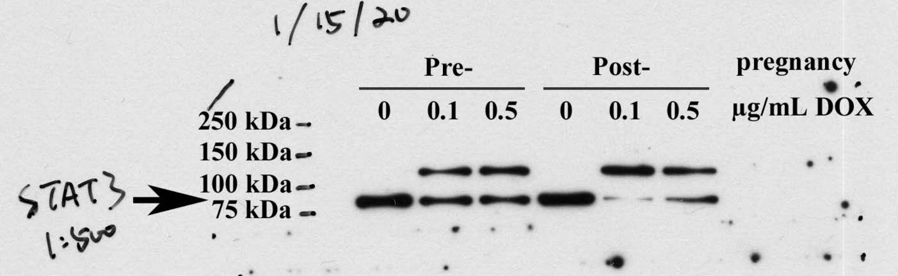

Feigman & Moss & Chen - STAT3 western blot - Sup.Fig.6i

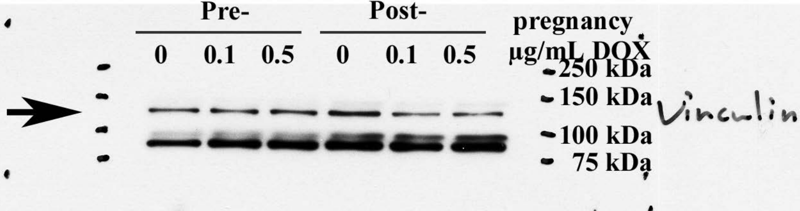

Feigman & Moss & Chen - Vinculin western blot - Sup.Fig.6i
